# Supplementary material for: Phenotype integration improves power and preserves specificity in biobank-based genetic studies of major depressive disorder
Source: Nat Genet. 2023 Nov 20;55(12):2082–93. doi: 10.1038/s41588-023-01559-9 (PMC10703686; doi:10.1038/s41588-023-01559-9)
Supplement: Supplementary file 2 — Reporting Summary [file 41588_2023_1559_MOESM2_ESM.pdf]

nature portfolio

Corresponding author(s): Na Cai, Andrew Dahl

Last updated by author(s): 04/12/2023

Reporting Summary

Nature Portfolio wishes to improve the reproducibility of the work that we publish. This form provides structure for consistency and transparency in reporting. For further information on Nature Portfolio policies, see our Editorial Policies and the Editorial Policy Checklist.

Statistics

For all statistical analyses, confirm that the following items are present in the figure legend, table legend, main text, or Methods section.

n/a	Confirmed	
☐	☒	The exact sample size (*n*) for each experimental group/condition, given as a discrete number and unit of measurement
☐	☒	A statement on whether measurements were taken from distinct samples or whether the same sample was measured repeatedly
☐	☒	The statistical test(s) used AND whether they are one- or two-sided<br>*Only common tests should be described solely by name; describe more complex techniques in the Methods section.*
☐	☒	A description of all covariates tested
☐	☒	A description of any assumptions or corrections, such as tests of normality and adjustment for multiple comparisons
☐	☒	A full description of the statistical parameters including central tendency (e.g. means) or other basic estimates (e.g. regression coefficient) AND variation (e.g. standard deviation) or associated estimates of uncertainty (e.g. confidence intervals)
☐	☒	For null hypothesis testing, the test statistic (e.g. *F*, *t*, *r*) with confidence intervals, effect sizes, degrees of freedom and *P* value noted<br>*Give P values as exact values whenever suitable.*
☒	☐	For Bayesian analysis, information on the choice of priors and Markov chain Monte Carlo settings
☒	☐	For hierarchical and complex designs, identification of the appropriate level for tests and full reporting of outcomes
☐	☒	Estimates of effect sizes (e.g. Cohen's *d*, Pearson's *r*), indicating how they were calculated

Our web collection on statistics for biologists contains articles on many of the points above.

Software and code

Policy information about availability of computer code

Data collection	No new data was collected for this study.
Data analysis	We conducted our analyses using the following published and publicly available software: 1) for computing PCA on all White British sample in UKBiobank: flashPCA v2.0 (https://github.com/gabraham/flashpca); 2) for GWAS using logistic regression: PLINK v2 (https://www.cog-genomics.org/plink2); 3) for estimation of heritability, genetic correlation and/or enrichment of heritability: LDSC v1.0.1 (https://github.com/bulik/ldsc); 4) for calculating polygenic risk scores: PRSice v2 (https://www.prsice.info/); 5) for imputing phenotypes in the MDD-related phenome: softImpute v1.4-1 (https://cran.r-project.org/web/packages/softImpute/index.html); 6) for imputing phenotypes in the MDD-related phenome with AutoComplete: https://github.com/sriramlab/AutoComplete; 7) for phenotype integration at the summary statistics level: MTAG v0.9.0 (https://github.com/JonJala/mtag); 8) for testing heterogeneity of effects between non-overlapping GWAS: METASOFT (http://genetics.cs.ucla.edu/meta/). We further provide custom code for running softImpute and calculating PRS Pleiotropy in https://github.com/andywdahl/mdd-impute and https://github.com/caina89/MDDImpute; this is reported in our Code Availability statement.

For manuscripts utilizing custom algorithms or software that are central to the research but not yet described in published literature, software must be made available to editors and reviewers. We strongly encourage code deposition in a community repository (e.g. GitHub). See the Nature Portfolio guidelines for submitting code & software for further information.

Data

Policy information about availability of data

All manuscripts must include a data availability statement. This statement should provide the following information, where applicable:

- Accession codes, unique identifiers, or web links for publicly available datasets
- A description of any restrictions on data availability
- For clinical datasets or third party data, please ensure that the statement adheres to our policy

We used the genotype and phenotype data from A) 502,637 samples in the full release (imputation version 2) of the UK Biobank Resource under application no. 28709 and 33217; B) iPSYCH cohorts 2012 and 2015i with genotype data and phenotype data on Major Depressive Disorder (2012: Ncontrols = 23,371, Ncases = 18,879; 2015i: Ncontrols = 15,163, Ncases = 8,188; Total: Ncontrols = 38,534, Ncases = 27,067); C) UCLA ATLAS electronic health record cohort where genotype data and phecode for depressive disorders and the subset Major Depressive Disorder were available (1,997 unrelated Asian-identifying individuals, 1,125 unrelated Black-identifying individuals, 2,169 unrelated Latino-identifying individuals, and 14,366 unrelated White-identifying individuals, see Supplemental Tables 5-6). Details of all cohorts used and both genotype quality control and phenotype selection are described in Supplementary Note. We further used publicly available summary statistics from other studies downloadable from the website of Psychiatric Genomics Consortium (https://www.med.unc.edu/pgc/results-and-downloads) and figshare for iPSYCH2012 (https://doi.org/10.6084/m9.figshare.20517330), and the references for which can be found in Supplemental Table 4. The individual-level CONVERGE, Danish and UCLA datasets are not publicly available due to institutional restrictions on data sharing and privacy concerns. We provide summary statistics of all GWAS described in this study on https://doi.org/10.6084/m9.figshare.19604335.

Human research participants

Policy information about studies involving human research participants and Sex and Gender in Research.

Reporting on sex and gender	We included self-reported sex/gender as a phenotype in our MDD-relevant phenome and the analysis of the latent factors from softImpute showed that sex/gender is reflected by one of the top factors accounting for variation in the MDD-related phenome (factor 5). We explain this in the manuscript. We further investigated if results of phenotype imputation differs if we stratified the data by sex and performed imputation separately in each sex; we found that the results of the sex-stratified imputation correlate highly with the joint-imputation, and for most phenotypes, including our focal phenotype LifetimeMDD, imputation does better when performed jointly on both sexes.
Population characteristics	We clearly describe characteristics of each studied cohort in our Methods and Supplementary Materials. In brief: (1) UK Biobank contains older British individuals (age range 37-73 at point of data collection), and we stratify our analyses using a combination of self-reported ethnicity and genetically-informed continental-level ancestry; (2) ATLAS contains diverse individuals in the UCLA medical system (age 18-86 at point of data collection); (3) iPSYCH contains Danish individuals who are drawn randomly from the population or have diagnoses of common mental disorders (age range 8-32 for iPSYCH2012, age range 8-35 for iPSYCH2015i, all ages at point of data collection); (4) CONVERGE contains Chinese women screened by mental health professionals (age range 30-60 at point of data collection).
Recruitment	No new data was collected for this study.
Ethics oversight	This research was conducted under the ethical approval from the UK Biobank Resource under application no. 28709 and 33217. The use of iPSYCH data follows standards of the Danish Scientific Ethics Committee, the Danish Health Data Authority, the Danish Data Protection Agency, and the Danish Neonatal Screening Biobank Steering Committee. Data access was via secure portals in accordance with Danish data protection guidelines set by the Danish Data Protection Agency, the Danish Health Data Authority, and Statistics Denmark. Retrospective data collection and analysis for ATLAS was approved by the UCLA IRB39. Patient Recruitment and Sample Collection for Precision Health Activities at UCLA is an approved study by the UCLA Institutional Review Board (UCLA IRB17-001013). All necessary patient/participant consent has been obtained and the appropriate institutional forms have been archived. The CONVERGE (China, Oxford, and VCU Experimental Research on Genetic Epidemiology) study was approved by the ethical review boards of Oxford University and participating hospitals. All participants provided written informed consent.

Note that full information on the approval of the study protocol must also be provided in the manuscript.

Field-specific reporting

Please select the one below that is the best fit for your research. If you are not sure, read the appropriate sections before making your selection.

☒ Life sciences ☐ Behavioural & social sciences ☐ Ecological, evolutionary & environmental sciences

For a reference copy of the document with all sections, see nature.com/documents/nr-reporting-summary-flat.pdf

Life sciences study design

All studies must disclose on these points even when the disclosure is negative.

Sample size	We used the genotype and phenotype data from A) 502,637 samples in the full release (imputation version 2) of the UK Biobank Resource

Sample size	under application no. 28709 and 33217; B) iPSYCH cohorts 2012 and 2015i with genotype data and phenotype data on Major Depressive Disorder (2012: Ncontrols = 23,371, Ncases = 18,879; 2015i: Ncontrols = 15,163, Ncases = 8,188; Total: Ncontrols = 38,534, Ncases = 27,067); C) UCLA ATLAS electronic health record cohort where genotype data and phecode for depressive disorders and the subset Major Depressive Disorder were available (1,997 unrelated Asian-identifying individuals, 1,125 unrelated Black-identifying individuals, 2,169 unrelated Latino-identifying individuals, and 14,366 unrelated White-identifying individuals, see Supplemental Tables 7-8). For all datasets, sample sizes were determined by the maximum number of individuals collected by the cohort with information on depression-related phenotypes rather than power analyses. Much of the work in this manuscript explores how much power these sample sizes have in GWAS and PRS analyses.
Data exclusions	We excluded all samples with 1) poor genotyping quality, 2) high level of relatedness to other samples, 3) ancestries other than White British as indicated by the QC metrics from UKBiobank (Bycroft et al 2018, https://doi.org/10.1038/s41586-018-0579-z), 4) sex chromosome aneuploidy, 5) withdrawal of consent from being included in research on data from the UKBiobank, 6) a history of substance abuse, and 7) manic or psychotic conditions. This gives us our final sample of 337,198 White-British, unrelated individuals. Details of exclusion criteria can be found in Supplemental Methods section "Sample filtering".
Replication	We replicated significant genetic effects identified in GWAS on imputed LifetimeMDD in UKBiobank (with both softImpute and Autocomplete) using summary statistics from external cohorts of MDD: PGC29, 23andMe and iPSYCH (this is described in Methods and references of the cohorts used can be found in Supplementary Table 4). We then replicated the improvement in PRS prediction accuracy in imputed and MTAG PRS, both in individuals of European ancestry and in other ancestries, in iPSYCH, ATLAS and CONVERGE.
Randomization	Not applicable, as no new assessments were performed in this study and all analyses were conducted on data from all individuals present in existing data.
Blinding	Not applicable, as data was collected by the time this study is conducted, all assessments on participants of cohorts used in this analyses were not blinded (if clinicians were involved, in CONVERGE, iPSYCH, UCLA ATLAS, and for all ICD codes in UKBiobank) or self-administered (for all self-reported depression related phenotypes and depression measures in UKBiobank).

Reporting for specific materials, systems and methods

We require information from authors about some types of materials, experimental systems and methods used in many studies. Here, indicate whether each material, system or method listed is relevant to your study. If you are not sure if a list item applies to your research, read the appropriate section before selecting a response.

Materials & experimental systems

n/a	Involved in the study
☒	☐ Antibodies
☒	☐ Eukaryotic cell lines
☒	☐ Palaeontology and archaeology
☒	☐ Animals and other organisms
☒	☐ Clinical data
☒	☐ Dual use research of concern

Methods

n/a	Involved in the study
☒	☐ ChIP-seq
☒	☐ Flow cytometry
☒	☐ MRI-based neuroimaging
